# Pre-operative stromal stiffness measured by shear wave elastography is independently associated with breast cancer-specific survival

**DOI:** 10.1007/s10549-018-4836-5

**Published:** 2018-06-01

**Authors:** Andy Evans, Yee Ting Sim, Celine Pourreyron, Alastair Thompson, Lee Jordan, Dawn Fleming, Colin Purdie, Jane Macaskill, Sarah Vinnicombe, Paul Pharoah

**Affiliations:** 1 Breast Imaging, Ninewells Hospital and Medical School, Mailbox 4, Level 6, Dundee, DD1 9SY UK; 2 Jackie Wood Cancer Centre, Ninewells Hospital and Medical School, Dundee, DD1 9SY UK; 3 Department of Breast Surgical Oncology, University of Texas MD Anderson Cancer Center, Houston, TX 77030 USA; 4 Pathology Department, Ninewells Hospital and Medical School, Dundee, DD1 9SY UK; 5 Breast Surgery, Ninewells Hospital and Medical School, Dundee, DD1 9SY UK; 6 Department of Public Health and Primary Care, Department of Oncology, Strangeways Research Laboratory, Worts Causeway, Cambridge, CB1 8RN UK

**Keywords:** Breast cancer, Prognosis, Shear wave elastography, Ultrasound, Neoadjuvant chemotherapy

## Abstract

**Introduction:**

With the increased use of neoadjuvant therapy for breast cancer, there is a need for pre-operative prediction of prognosis. We aimed to assess the prognostic value of tumour stiffness measured by ultrasound shear wave elastography (SWE).

**Methods:**

A consecutive cohort of patients with invasive breast cancer underwent breast ultrasound (US) including SWE. The following were recorded prospectively: US diameter, stiffness at SWE, presentation source, core biopsy grade, oestrogen receptor (ER) status and pre-operative nodal status. Breast cancer-specific survival (BCSS) was analysed with regard to US size and stiffness, tumour grade on core biopsy, ER status, presentation mode and pre-operative nodal status. Analysis used Cox proportional hazards regression.

**Results:**

Of the 520 patients, 42 breast cancer and 53 non-breast cancer deaths were recorded at mean follow-up of 5.4 years. Hazard ratios (HR) for tertiles of stiffness were 1, 4.8 and 8.1 (*P* = 0.0001). HR for 2 groups based on US size < or ≥ 20 mm were 1 and 5.1 (*P* < 0.0001). HR for each unit increase in tumour grade on core biopsy was 3.9 (*P* < 0.0001). The HR for ER positivity compared to ER negativity was 0.21 (*P* < 0.001). BCSS was also associated with presentation mode and pre-operative nodal status. In a multivariable model, stiffness, US size and ER status were independently associated with BCSS.

**Conclusion:**

Multiple pre-operative factors including stromal stiffness at SWE have independent prognostic significance. A larger dataset with longer follow-up could be used in the future to construct a pre-operative prognostic model to guide treatment decisions.

## Introduction

The assessment of prognosis has traditionally been performed after surgical excision of breast cancer using the classical prognostic factors of invasive tumour size, lymph node status, histological grade and vascular invasion status [1]. These prognostic factors are then used to guide decisions regarding adjuvant systemic therapy. In recent years, the immunophenotype [2] and molecular phenotype [3] have also been used to inform these decisions. Online resources, such as Predict, are also widely used to give information on overall survival and the possible benefits of adjuvant therapy. However, with the increased use of neoadjuvant chemotherapy (NACT) there is a need for accurate pre-operative prediction of prognosis to aid treatment selection. Pre-operatively, tumour size can be estimated from imaging and histological grade and ER and HER-2 status determined from the core biopsy [4]. Mode of presentation (through mammographic screening or symptoms) has also been shown to affect outcome even when adjusted for pathological variables such as invasive tumour size and nodal status [5]. Ultrasound of the axilla with biopsy of abnormal nodes is diagnostic for approximately 50% of node-positive patients [6]. However, given the debate regarding the need for clearance of all positive axillae, vigorous efforts to diagnose every positive axilla pre-operatively are being discouraged by some surgeons [7]. There is, therefore, a need for more reliable tools for the pre-operative assessment of prognosis.

The tumour microenvironment greatly contributes to cancer growth, dissemination and drug resistance. One of the key features involved in these functions is the extracellular matrix (ECM) remodelling. This process is characterised by an increased number of stromal cells, an increased secretion of extracellular matrix proteins as well as thickening and reorganization of the collagen fibrils which results in stiffening of the stroma [8]. A biophysical and histological study has shown that stromal stiffness is higher at the invasive front of the most aggressive breast tumours (HER2 amplified and triple-negative breast cancer, TNBC) compared to the less aggressive luminal tumours, suggesting an association between stiff stroma and cancer aggression [9]. Conklin et al. have correlated collagen fibril orientation with poor survival regardless of tumour grade, size, node status and tumour subtype in breast cancer [10]. In vitro studies have demonstrated that substrate stiffness mediates drug resistance in breast cancer cell lines [11]. In the neoadjuvant setting, a stromal gene signature enriched in ECM protein was found to be associated with poor response to 5-fluorouracil, epirubicin and cyclophosphamide treatment [12]. All these findings led us to investigate stromal stiffness measured by shear wave elastography (SWE) as a prognostic marker of breast cancer survival. Shear wave elastography (SWE) is an ultrasound imaging method which allows quantification of lesional and peri-lesional stiffness and it has been shown to aid benign/malignant differentiation of breast masses [13]. SWE is highly reproducible and quantitative [14]. Both stiffness at SWE and strain elastography have been shown to be predictors of nodal metastasis, independent of invasive tumour size, histological grade and vascular invasion status [15, 16]. Stiffness at SWE has a strong relationship with invasive tumour size, histological grade and poor outcome immunophenotypes [17, 18]. Stiffness at elastography has also been shown, in some studies, to be associated with chemotherapy resistance [19–21].

We therefore postulated that stiffness at SWE may be related to the prognosis of women with breast cancer. No previous studies have addressed this question. The aim of the study is to investigate associations between prospectively collected pre-operative factors (including stromal stiffness) and breast cancer survival in a consecutive cohort of women diagnosed with ultrasound visible, invasive breast cancer.

## Methods

SWE, US, source of referral and histopathological details (including core biopsy grade, ER, HER-2 status and pre-operative nodal status) were collected prospectively from a consecutive series of patients undergoing diagnostic breast examination for lesions subsequently shown to be invasive breast cancer (*n* = 540). Between April 2010 and January 2013, all women had their US lesion diameter and mean stiffness (kPa) at SWE recorded irrespective of subsequent treatment (primary surgery, neoadjuvant systemic therapy and primary endocrine therapy). The core biopsy grade was recorded to allow inclusion of this parameter in assessment of prognosis pre-operatively. ER status and HER-2 status are routinely measured in our institution on the core biopsy rather than the surgical resection specimen in line with current guidelines. All women had axillary US and core biopsy of abnormal nodes (cortex > 2.3 mm) for assessment of nodal status.

All US scans were performed by one of five breast radiologists or an advanced radiography practitioner trained to perform and interpret breast ultrasound. These practitioners had between 7 and 22 years of breast ultrasound experience and had at least 12 months of experience performing SWE of solid breast lesions. Four SWE images in two orthogonal planes were obtained. The region of interest (ROI) utilized in all cases was 2 mm in diameter. Mean stiffness in kPa was taken as the average of the values taken from four SWE images taken in two orthogonal planes. The maximum US diameter used in the analysis was the largest obtained in any of the three planes. All scans were performed using an Aixplorer® ultrasound system (SuperSonic Imagine, Aix en Provence, France). Institutional Review Board ethical approval was waived for this retrospective analysis of prospectively recorded data and all patients gave permission for evaluation of their images.

Patient’s survival including cause of death was ascertained from local paper and electronic health records and the National Cancer Registry. Patients that died after developing metastatic breast cancer were assumed to have died of breast cancer. A total of 20 patients were excluded from the analysis on the following grounds: Twelve patients with metastases at presentation; five where cause of death could not be ascertained; two with no follow-up data; and one with a history of a previous breast cancer.

BCSS was assessed using Kaplan–Meier survival curves. Association between putative prognostic variables and BCSS was evaluated using Cox proportional hazards regression. Variables included in Cox models were mean stiffness, tumour size, tumour grade, ER status, presentation source (screening or symptomatic) and pre-operative nodal status. Mean stiffness was categorized into three equal size groups and size into two groups (< 20 or ≥ 20 mm).

## Results

After the 20 exclusions detailed above, 520 patients constituted the study group (mean age 62 years, median age 62 years and range 28–95 years). Two hundred and five (39%) patients had their cancer diagnosed at mammographic screening while 315 (61%) women had symptomatic cancers. The pathological characteristics of the tumours are shown in Table 1. Four hundred and twenty-one women underwent immediate surgery and surgical nodal staging of whom 42% had invasive cancers ≥ 20 mm in size and 29% had axillary macro-metastases. Of the remaining 99, four women had immediate surgery but did not undergo nodal staging. Forty-five women were treated with NACT and 11 had neoadjuvant endocrine therapy. Thirty-nine women were treated using primary endocrine therapy due to severe co-morbidities. The mean follow-up in women still alive at the time of reporting was 5.4 years.

**Table 1:** Pathological and immunohistochemical characteristics of study cancers

Histological grade 1	60 (12%)
Histological grade 2	225 (43%)
Histological grade 3	235 (45%)
Ductal carcinoma of no specific type	399 (77%)
Lobular cancer	65 (13%)
Tubular cancer	19 (4%)
< 10 mm	57 (13%)
10–20 mm	189 (44%)
21–30 mm	103 (24%)
> 30 mm	76 (18%)
Vascular invasion	108 (25%)
Node positive (macro-metastases)	122 (29%)
ER positive	429 (83%)
PR positive	373 (72%)
HER-2 positive	63 (12%)

As not all patients had immediate surgery denominators for vascular invasion, nodal status and invasive size vary

**Fig. 1:**
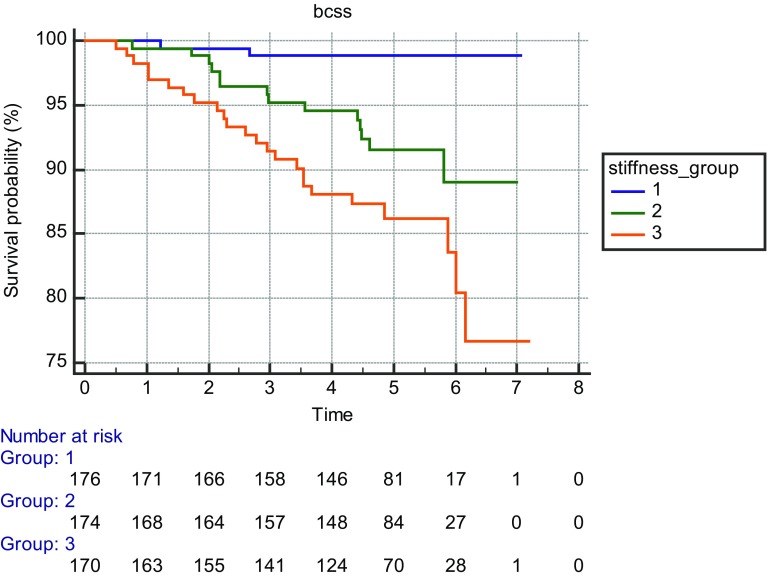
Kaplan-Meier survival curves for three equal size groups based on stiffness at shear wave elastography

**Fig. 2:**
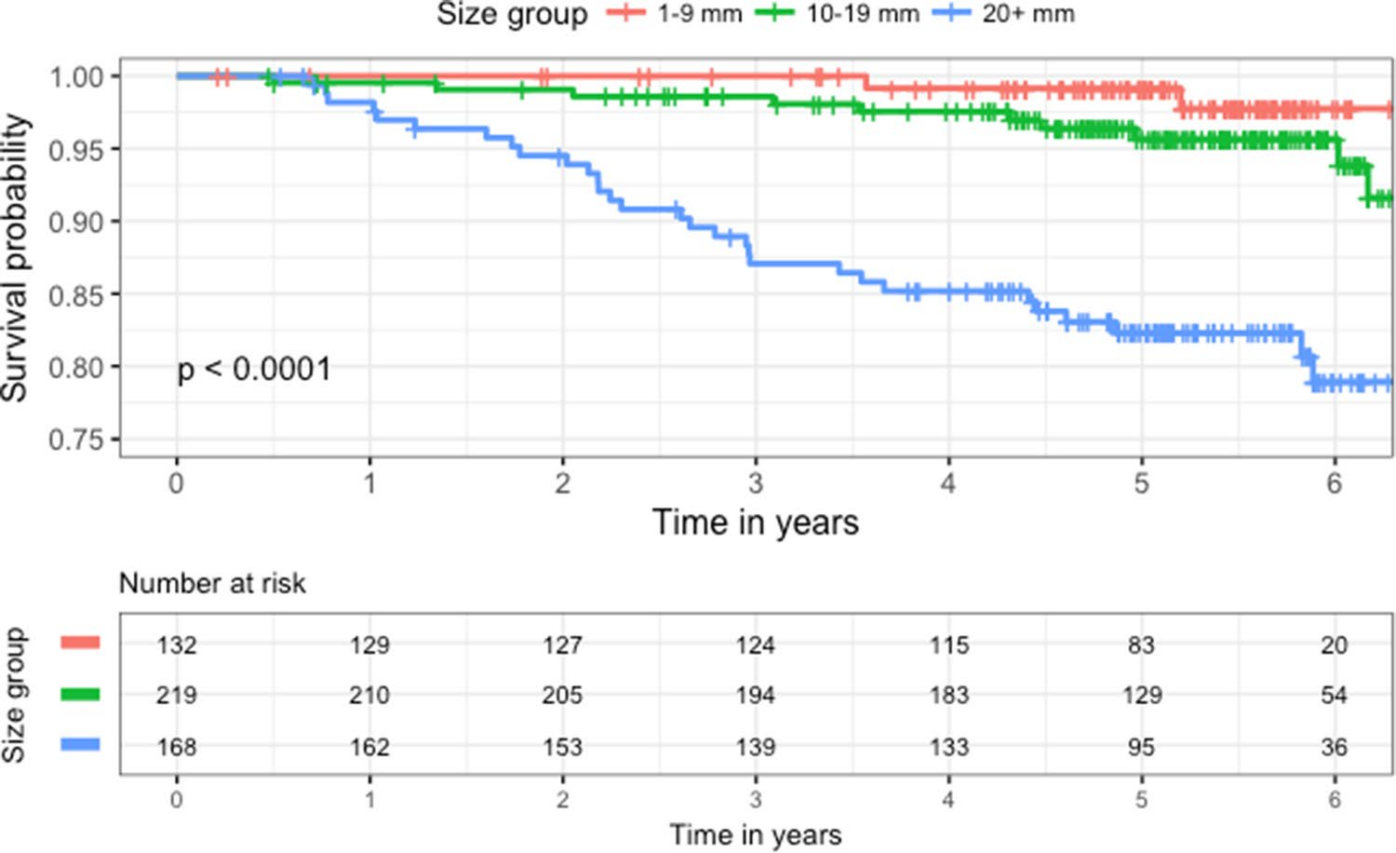
Kaplan-Meier survival curves according to ultrasound size

**Fig. 3:**
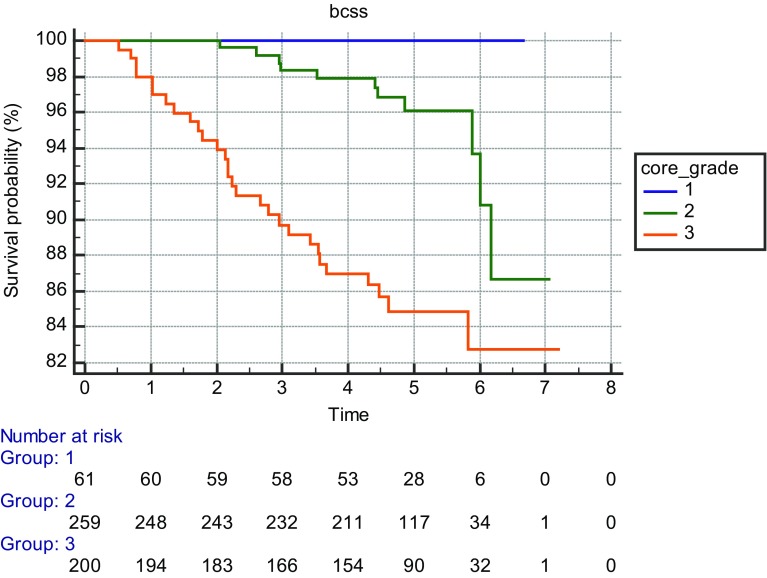
Kaplan-Meier survival curves according to core biopsy estimated histological grade

Forty-two women of the 520 died of breast cancer (8%) while 53 women had non-breast cancer deaths (10%) during the follow-up period. Kaplan–Meier survival curves for three equal size groups based on stiffness are shown in Fig. 1 and curves by US size in Fig. 2. Kaplan–Meier survival curves according to core biopsy grade are shown in Fig. 3.

All variables except HER-2 were significantly associated with BCSS in univariable Cox regression models (Table 2). However, in a multivariable model, grade and mode of detection were no longer significant at a nominal *P* < 0.1 and these variables were excluded from the final multivariable model (Table 2). In this model, stiffness was strongly associated with BCSS (tertile 2 vs tertile 1 HR = 3.4, 95% CI 0.95–12 and tertile 3 vs tertile 1 HR = 4.7, 95% CI 1.4–16).

**Table 2:** Results of univariable and multivariable analyses of pre-operative prognostic markers

	Univariable model			Multivariable model			Final multivariable model		
	HR	95% CI	*P* value	HR	95% CI	*P* value	HR	95% CI	*P* value
Stiffness									
Tertile 2 vs 1	4.9	1.4–17	0.013	2.7	0.76–9.8	0.12	3.4	0.95–12	0.06
Tertile 3 vs 1	8.2	2.5–27	0.0006	4	1.2–14	0.028	4.7	1.4–16	0.013
Size									
20+ mm vs < 20 mm	5.4	2.8–11	8 × 10^− 7^	2.8	1.4–5.7	0.0023	3.5	1.8–7.0	0.00033
Core grade	3.9	2.1–7.2	2 × 10^− 5^	1.8	0.83–3.8	0.14			
Pre-op node positive	2.9	1.5–5.6	0.0014	1.8	0.90–3.6	0.096	2	1.0–3.9	0.047
ER positive	0.21	0.11–0.38	4 × 10^− 7^	0.31	0.15–0.63	0.0014	0.23	0.13–0.43	3 × 10^− 6^
Screen vs clinical detection	5.1	2.0–13	0.00065	1.7	0.64–4.7	0.28			
HER-2	1.3	0.55.–3.11	0.55	0.63	0.26–1.5	0.31			

## Discussion

We have shown that US size, SWE stiffness, ER status and pre-operative nodal status are independent pre-operative predictors of prognosis in invasive breast cancer. The other pre-operative factors studied (core grade, and presentation) failed to reach statistical significance at multivariate analysis but given the small number of events (*n* = 42) this could reflect the limited power of the study rather than true lack of effect.

If these factors are shown to be prognostic in larger and independent datasets, then a pre-operative prognostic model could be constructed and validated in the future. Such a pre-operative prognostic model could also be used to guide clinical decisions such as the appropriateness of NACT. Current prognostic models such as the NPI [22] and PREDICT use post-operative factors so cannot be used pre-operatively.

Grey scale US is used globally to evaluate breast masses, while SWE has gained use in recent years, and is now available from many leading equipment manufacturers. Performing SWE takes about 2 min per patient and reading the stiffness from the acquired images also takes 2 min per patient [23]. In this study, we used *E*_mean_ measurements but *E*_max_ measurement give equally good results in studies of benign/malignant differentiation [24]. Including SWE in the routine imaging examination of solid breast masses would therefore not be difficult to introduce in most breast clinics. Core biopsy ER status is also routinely available in nearly every case of both screening and symptomatic invasive cancer.

The stiffness within breast cancers at SWE is predominantly seen at the tumour/stromal boundary and in the peri-tumoural stroma [9, 16, 23]. This stiffness appears to be due to the nature of the collagen produced by tumour-associated stromal cells (fibroblasts and cancer-associated fibroblasts (CAFs)) [25]. An increase in stromal stiffness induces activation of the CAFs into myofibroblasts expressing aSMA [26] and SNAIL1 [27] resulting in higher contractibility capacities and maintenance of ECM protein secretion that further contributes to tissue stiffening. Indeed, CAFs have been shown in vitro to cause an eightfold increase in matrix stiffness compared with normal fibroblasts through the production of thicker collagen and increased collagen cross linking by lysyl oxidase [27, 28]. This process results in the release of active TGFb which also maintains CAFs into their active form [29].

As well as being a risk factor for breast cancer development, stiffening of the tumour stroma has been shown to enhance several key functions of tumour development. It does so by triggering mechano-responses of the tumour cells through mechano-sensors such as integrins [30]. More particularly, in vivo and in vitro breast models have shown that activation of the focal adhesion kinase (FAK), in response to stiff tissue, promotes tumour cell proliferation and an invasive phenotype [31]. Moreover, perpendicular orientation of the collagen bundles to the primary lesion observed in stiff tissue allows tumour cells migration towards blood vessels as well as myeloid cell infiltration [14]. Tumour cell dissemination is also encouraged by the increased vascularisation which develops in response to stiffness-associated hypoxia. Stiff ECM also induces drug resistance by limiting therapeutic agent diffusion and by activating cellular pathways involved in tumour cell survival [11].

This non-exhaustive list of the pro-tumoural effects of stiff stroma surrounding breast tumour cells provides clues as to why stiff tissue measurement by shear wave elastography is a prognostic marker of breast cancer patient survival.

Breast MRI, particularly diffusion-weighted imaging has been shown to correlate both with prognostic factors such as tumour size, histological grade and nodal positivity [32, 33] and in combination with other imaging parameters may improve pre-operative prognostication.

Core biopsy ER status is routinely used to guide decisions regarding pre-operative therapy and is preferred by many to ER status derived from the surgical specimen due better fixation of the small sample.

The four factors found to be independently significant are therefore based on the biology of the tumour (ER status), the tumour microenvironment (stiffness) and the time-dependent variables of lesion size (as measured by ultrasound) and pre-operative nodal status.

Lymph node status is, historically, the most powerful of the classical prognostic factors [1] but pre-operative nodal status was only weakly independently significant in this study. While US size and stiffness have been previously shown to have independent associations with nodal positivity, the present model reflects the cancer biology (of the tumour and the microenvironment) upon which the traditional anatomical TNM size-based criteria (including tumour size and node status) are predicated. The current debate regarding the need for surgical treatment of the positive axilla means aggressive pre-operative diagnosis of positive axillae is not welcomed by some surgeons and oncologists [7]. Therefore, having a prognostic model which is not heavily reliant on axillary staging may be seen by some as advantageous. The combination of diffusion-weighted MR imaging with morphological and dynamic MR imaging findings might be used in the future for differentiation of metastatic from benign axillary lymph nodes without the need for biopsy [34].

The current study was carried out in a single centre with a longstanding research interest in SWE. However, SWE is an easy technique to learn and has been shown to have excellent reproducibility [14] and is in use globally. Clearly, before a practice change can be adopted into routine clinical practice, the findings of this study require confirmation in an independent series with prospective validation.

In conclusion, we have found that US size, stiffness, ER status and pre-operative nodal status are pre-operative factors which have independent prognostic significance for breast cancer-specific survival. Given larger numbers and longer follow-up, a simple, practical model could be constructed and used to assess the appropriateness of neoadjuvant systemic therapy and inform patients who wish to know prognostic information prior to surgery.
